# A genome wide pattern of population structure and admixture in peninsular Malaysia Malays

**DOI:** 10.1186/s11568-014-0005-z

**Published:** 2014-10-30

**Authors:** Wan Isa Hatin, Ab Rajab Nur-Shafawati, Ali Etemad, Wenfei Jin, Pengfei Qin, Shuhua Xu, Li Jin, Soon-Guan Tan, Pornprot Limprasert, Merican Amir Feisal, Mohammed Rizman-Idid, Bin Alwi Zilfalil, The HUGO Pan-Asian SNP Consortium

**Affiliations:** 1 Human Genome Centre, School of Medical Sciences, Universiti Sains Malaysia, 16150 Kelantan, Malaysia; 2 Department of Pediatrics, School of Medical Sciences, Universiti Sains Malaysia, 16150 Kelantan, Malaysia; 3 Chinese Academy of Sciences and Max Planck Society (CAS-MPG) Partner Institute for Computational Biology, Shanghai Institutes for Biological Sciences, Chinese Academy of Sciences, 200031 Shanghai, China; 4 Department of Cell and Molecular Biology, Faculty of Biotechnology and Biomolecular Sciences, Universiti Putra Malaysia, 43400 Selangor, Malaysia; 5 Human Genetics Unit, Department of Pathology, Faculty of Medicine, Prince of Songkla University, Hat Yai, Songkhla, 90110 Thailand; 6 Institute of Biological Sciences, 50603 Kuala Lumpur, Malaysia; 7 Centre of Research for Computational Sciences and Informatics in Biology, Bioindustry, Environment, Agriculture and Healthcare (CRYSTAL),Faculty of Science, Universiti Malaya, 50603, Kuala Lumpur, Malaysia

**Keywords:** Malays, Single nucleotide polymorphisms, Genetic structure, Admixture, Haplotypes

## Abstract

**Background:**

The Malays consist of various sub-ethnic groups which are believed to have different ancestral origins based on their migrations centuries ago. The sub-ethnic groups can be divided based on the region they inhabit; the northern (*Melayu Kedah* and *Melayu Kelantan*), western (*Melayu Minang*) and southern parts (*Melayu Bugis* and *Melayu Jawa*) of Peninsular Malaysia. We analyzed 54,794 autosomal single nucleotide polymorphisms (SNPs) which were shared by 472 unrelated individuals from 17 populations to determine the genetic structure and distributions of the ancestral genetic components in five Malay sub-ethnic groups namely *Melayu Bugis*, *Melayu Jawa*, *Melayu Minang*, *Melayu Kedah*, and *Melayu Kelantan*. We also have included in the analysis 12 other study populations from Thailand, Indonesia, China, India, Africa and *Orang Asli* sub-groups in Malay Peninsula, obtained from the Pan Asian SNP Initiative (PASNPI) Consortium and International HapMap project database.

**Results:**

We found evidence of genetic influx from Indians to Malays, more in *Melayu Kedah* and *Melayu Kelantan* which are genetically different from the other Malay sub-ethnic groups, but similar to Thai *Pattani*. More than 98% of these northern Malays haplotypes could be found in either Indians or Chinese populations, indicating a highly admixture pattern among populations. Nevertheless, the ancestry lines of Malays, Indonesians and Thais were traced back to have shared a common ancestor with the Proto-Malays and Chinese.

**Conclusions:**

These results support genetic admixtures in the Peninsular Malaysia Malay populations and provided valuable information on the enigmatic demographical history as well as shed some insights into the origins of the Malays in the Malay Peninsula.

## Background

The knowledge of population genetic structure and genetic ancestry hold great potential towards better understanding of the differential susceptibility to disease, response to drugs and complex interaction of genetic and environment factors (Collin et al. 2003; Campbell and Tishkoff 2008). Recent studies have highlighted the importance of characterizing the genetic make-up of admixed populations (Sankararaman et al. 2008; Bryca et al. 2010 and Patterson et al. 2010. The admixtures within individual may affect the interactions between complex genes with other genes and environmental factors, and at the same time, also affect the susceptibility of individual to particular diseases (Collin et al. 2003; Tang et al. 2005; Lao et al. 2006 and Hunley et al. 2009). In addition, the analysis of genetic variations also provides detail knowledge for understanding the ancient human demographic history. The enigmatic history of Malays as well as their morphological features that exhibit fusion from various ethnicities and cultural background (Rahman 1998; Andaya 2001; Reid 2001; Hussein et al. 2007 and Omar 2004) have made them a uniquely complex population and intriguing subject to be studied.

In Peninsular Malaysia, the Malays form the majority of the population (63.1%) followed by Chinese (24.6%) and Indians (7.3%) (Jabatan Perangkaan Malaysia 2010). The intermarriage and integration among them for centuries had given complex admixtures in genome of Malays. Moreover, the Malays also known to have various sub-ethnic groups due to different ancestral origins based on their migrations centuries ago (Paul 1961). Thus, it is important to understand the definition of Malays either sociologically or anthropologically, in order to select the sampling populations which were relevant to the aim of this study. Sociologically, Malays are Malaysian citizen born to a Malaysian citizen who professes the religion of Islam, habitually speaks the Malay language, conforms to Malay custom and is domiciled in Malaysia (Constitution of Malaysia). Anthropologically, the Malays are described as an ethnic group of Austronesian people who speak Malayo-Polynesian language that belong to the Southern Mongoloid group of races and predominantly inhabit the Malay Peninsula (comprises of southern Thailand, Peninsular Malaysia, and the island of Singapore), south coast of Myanmar, eastern Sumatra, the coast of Borneo and the smaller islands between these locations - collectively known as the *Alam Melayu*. These locations today are part of the modern nations of Malaysia (Peninsular and Eastern Malaysia), Indonesia, Singapore, Brunei, Southern Myanmar and Southern Thailand (Omar 2004; Bellwood 1997). The existences of indigenous *Orang Asli* (aboriginal peoples) populations in the Peninsular Malaysia such as the *Semang* and Proto-Malays have also raised questions as to what extent they have contributed to the uniquely admixed gene pool of Malays (Bellwood 1993). The relationship between the Malays and the *Orang Asli*, especially with the *Semang* who are believed to be the earliest settlers and original coastal inhabitants of the Malay Peninsula (Allen 1879; Carey 1976 and Fix 1995) were important to be studied in order to identified the origin of Malays as well as the occupancy of prehistoric human populations in this region.

The previous study has shown that there is genetic substructure among Malays (Hatin et al. 2011). The *Melayu Kelantan* in north-eastern regions was genetically different from other Malay populations in the western (*Melayu Minang*) and southern parts (*Melayu Jawa* and *Melayu Bugis*) of the Peninsular Malaysia (Hatin et al. 2011). Beside, close genetic relationship of the *Melayu Kelantan* with the Indians and the *Orang Asli Semang* (*Jahai* and *Kensiu*) was also established (Hatin et al. 2011). Against these backgrounds, we conducted this study to investigate the extent of admixture in Malays, especially in northern Malays of Peninsular Malaysia using a model-based clustering method. The model-based methods attempt to more directly reconstruct historical events. This method is computationally intensive but it is explicit where the assumptions are stated, not hidden. In addition, we performed haplotype sharing analysis to consider the question of whether any outlier is migrants, experienced admixture or ancient population. Therewith, we included two more populations from northern part of peninsula, which are *Melayu Kedah* and Thai *Pattani* to verify the divergence pattern of the northern Malays.

## Results

### Pattern of genetic variations among populations

The genetic variations within and between five Peninsular Malaysia Malay sub-ethnic groups and other studied populations were characterized by the pair-wise Fst between populations, followed by the non-parametric Multi-Dimensional Scale (MDS) analysis. The table of pair-wise Fst value that has been multiplied with 1000 is shown in Table 1. All of the genetic distance values that showed closer relationship between populations were shaded in gray color. The genetic divergence between five of the Peninsular Malaysia Malay sub-ethnic groups shows significant difference of the *Melayu Bugis* from the other Malays which is substantially closer to Indonesian *Toraja* (Fst = 0.019).

**Table 1:** **Pair-wise Fst (x 1000) between the Malay sub-ethnic groups and other populations in this study**

	**MY-BG**	**MY-JV**	**MY-MN**	**MY-KN**	**MY-KD**	**TH-PT**	**MY-TM**	**MY-JH**	**MY-KS**	**ID-JV**	**ID-ML**	**ID-TR**	**CN-JN**	**CN-WA**	**IN-WL**	**IN-DR**	**YRI**
MY-BG																	
MY-JV	24																
MY-MN	24	21															
MY-KN	23	**19**	**18**														
MY-KD	22	**18**	**18**	**15**													
TH-PT	26	21	23	21	**18**												
MY-TM	26	**19**	**21**	**18**	**18**	**23**											
MY-JH	42	34	35	32	31	37	30										
MY-KS	53	47	46	42	41	48	41	23									
ID-JV	22	**16**	**19**	**17**	**17**	**21**	**17**	**32**	44								
ID-ML	26	25	23	23	23	28	25	41	53	23							
ID-TR	**19**	22	21	21	21	25	23	40	52	20	22						
CN-JN	31	25	27	24	23	28	25	40	52	23	31	28					
CN-WA	24	**18**	**20**	**17**	**17**	**21**	**17**	**32**	44	**15**	24	22	17				
IN-WL	57	54	46	**42**	**39**	48	50	57	61	52	57	56	57	51			
IN-DR	51	48	40	**36**	**33**	42	44	50	55	46	51	50	51	45	17		
YRI	112	109	102	99	97	106	104	111	116	107	112	110	112	106	88	84	

*The bold numbers indicate close genetic relationship between the populations.

*Melayu Kelantan* and *Melayu Kedah* were genetically close to each other (Fst = 0.015). Meanwhile, the genetic divergence between *Melayu Jawa* and *Melayu Minang* (Fst = 0.021) showed that they were closer to *Melayu Kedah* and *Melayu Kelantan* than to each other. Interestingly, these four Malay sub-ethnic groups were significantly closer to Proto-Malays *Temuan*, Indonesian *Jawa* and Chinese *Wa* from Yunnan, China. The genetic distances between the Proto-Malays *Temuan*, Indonesian *Jawa*, and Chinese *Wa* to each other were also substantially lower than to any other populations, even between population within their groups. Although the Thai *Pattani* samples were also close to these group, but they were much closer to *Melayu Kedah* relative to the other Peninsular Malaysia Malays (Fst = 0.018).

In relation with the *Semang* group, both the *Melayu Kedah* and *Melayu Kelantan* samples were relatively closer to the *Jahai* and *Kensiu* than any other Malays. Similarly, both the *Semang* samples were closer to the samples of Indonesian *Jawa* and Chinese *Wa* than any other populations. It is also noted that the genetic distance between *Melayu Kedah* and *Melayu Kelantan* with the Indians, especially with *Telugu* were considerable smaller than to any other populations.

**Figure 1:**
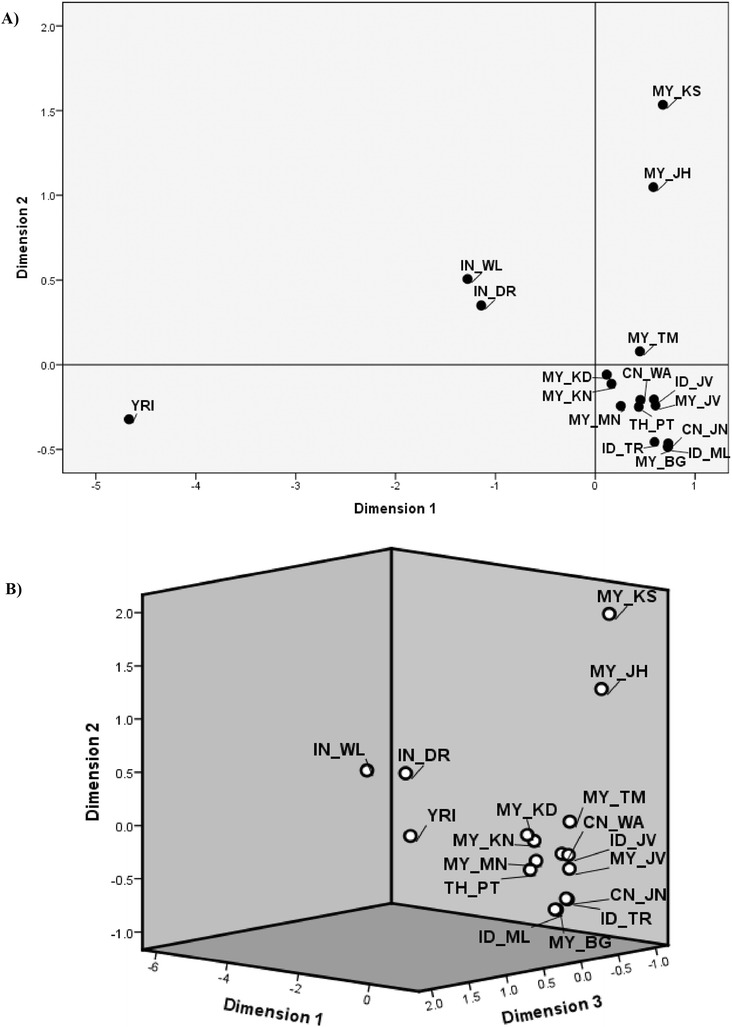
**MDS analysis for 17 populations based on Fst. A)** two dimensions (2D) and **B)** three dimensions (3D).

The MDS analysis for 17 populations was performed in two dimensions (2D) and three dimensions (3D) based on Fst genetic distance method as shown in Figure 1. The genetic variation of Malays showed by the pair-wise Fst was recaptured by the MDS scatter plot. The MDS scatter plot in 2D platform (Figure 1A) exhibited that all the Peninsular Malaysia Malay, Indonesian, Thai, Proto-Malay and Chinese populations were scattered closely at the below-right corner of the plot, which are near to the intersection of axes. They were far separated from three other group populations which are *Yoruba*, Indians and *Semang* that are far more diversified than the modern Malays. The same pattern also can be seen in the 3D MDS plot (Figure 1B) where all five Peninsular Malaysia Malay sub-ethnic groups were well separated into three different sub-clusters, although they still remained in the same dimensional platform (dimension 3) indicating an existence of substructure within the Peninsular Malaysia Malays. The MDS analysis showed that there were possible admixtures among *Melayu Kedah*, *Melayu Kelantan*, *Melayu Minang* and Thai *Pattani*. In the case of *Orang Asli* group, the genetic structure clearly appeared in the Proto-Malay *Temuan* with possible admixture to *Jawa* populations and Chinese *Wa*.

### Population genetic structure and ancestry

**Figure 2:**
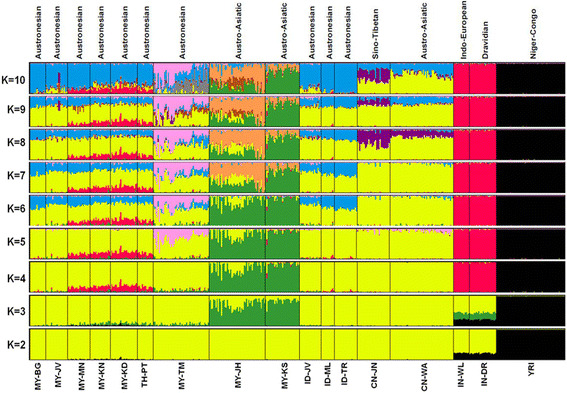
**The estimated population structure and ancestral membership coefficients of each of the 472 individuals for K = 2 to K = 10 from dataset S2.** The linguistic family of populations were showed at the top of the figure while the name of populations were showed below the figure. Each population was separated by the solid line and each individual was represented by a thin vertical line, which was partitioned into *K* color segment that represent the individual’s estimated *Q* fractions in *K* clusters.

The assignment of each of the 472 individuals sampled from 17 pre-defined populations into genetically inferred clusters of *K* = 2 to *K* = 10 is shown in Figure 2. Each individual was represented by a thin vertical line, which was partitioned into *K* color segment that represent the individual’s estimated *Q* fractions in *K* clusters. Each population was labeled below the figure and separated by the solid line. The results showed that individuals from the same pre-defined population shared almost similar *Q* values. Any pre-defined populations which shared similar distinctive *Q* values were merged into inferred cluster, as shown in Table 2.

**Table 2:** **Proportion of membership coefficient (** ***Q*** **) for each of population in each of the six inferred clusters (** ***K*** **= 6)**

**Country & Ethnicity (Sample size)**	**Location/State**	**Population ID**	**Clusters (** ***K*** **= 6)**					
			**Malays**	**Proto-Malays**	***Semang***	**Chinese**	**Indians**	**African**
Malaysia:								
Malays*:								
Jawa (19)	Johor	MY-JV	0.419	0.004	0.010	0.665	0.004	0.001
Bugis (14)	Johor	MY-BG	0.255	0.027	0.044	0.561	0.008	0.001
Minang (20)	Negeri Sembilan	MY-MN	0.318	0.011	0.019	0.525	0.125	0.002
Kelantan (18)	Kelantan	MY-KN	0.222	0.018	0.054	0.542	0.162	0.002
Kedah (24)	Kedah	MY-KD	0.206	0.028	0.031	0.527	0.208	0.001
Proto-Malay^a^:								
Temuan (49)	Negeri Sembilan	MY-TM	0.101	0.361	0.053	0.478	0.006	0.001
Negritos^a^:								
Jahai (50)	Perak	MY-JH	0.012	0.010	0.808	0.168	0.002	0.000
Kensui (30)	Kedah	MY-KS	0.018	0.006	0.926	0.035	0.015	0.001
Thailand*:								
Pattani (14)	Pattani	TH-PT	0.237	0.013	0.039	0.570	0.140	0.001
Indonesia^a^:								
Jawa (19)	Java	ID-JV	0.251	0.029	0.048	0.663	0.008	0.001
Melayu (12)	Sumatera	ID-ML	0.378	0.005	0.018	0.586	0.012	0.001
Toraja (20)	Sulawesi	ID-TR	0.444	0.004	0.009	0.540	0.003	0.001
China^a^:								
Jinuo (29)	Yunnan	CN-JN	0.016	0.005	0.015	0.959	0.005	0.001
Wa (56)	Yunnan	CN-WA	0.032	0.005	0.021	0.936	0.005	0.001
India^a^:								
Marathi (14)	Maharashtra	IN-WL	0.002	0.003	0.006	0.006	0.979	0.004
Telugu (24)	Andra Pradesh	IN-DR	0.003	0.003	0.011	0.006	0.975	0.002
Africa^b^:								
Yoruba (60)	Nigeria	YRI	0.001	0.001	0.003	0.001	0.002	0.992

^a^The genotype data obtained from the database of PASNPI Consortium (http://www4a.biotec.or.th/PASNP/).

^b^The genotype data obtained from International HapMap Consortium (http://hapmap.ncbi.nlm.nih.gov/).

*The inclusion criteria are; the sampled individual of a population must be at least three generations of the same population, no parental admixture and communicate daily in the local dialect. The exclusion criteria are those that contradict the inclusion criteria.

The most probable number of ancestral clusters was determined by the maximum value of the *Ln(Pr)* of *K* and by careful observation and comparison of each of the *Q*s of *K*s from multiple runs between the same and different sampling datasets using the SSC. The value of *Ln(Pr)* was observed to increase until *K* = 6 and started to become relatively inconsistent at *K* = 7 onwards. The SSC scores were greater than 0.95 in all cases of *K* < 6 while for larger *K*s (*K* > 5) the SSC were slightly lower with a minimal value of 0.90. In analysis of *K* > 6, the splitting orders of clusters varied across different runs and different datasets. However, for the same cluster mode, the SSC of membership coefficient estimates were still high (>0.90). Therefore, the *K* = 6 was considered as the most statistically supported by the data in all of the sampling datasets, depicted by six different colors in such a way as that given by the *Q* value (Table 2), corresponds to the fraction of genome inferred to have ancestry in the cluster.

At *K* = 2, all samples were separated into two distinct cluster of African (*Yoruba*) in black color, and non-African populations that were grouped into a yellow colored cluster. At *K* = 3, a newly cluster in green color represent *Seman*g samples from Peninsular Malaysia (*Jahai* and *Kensiu*). The green component of the *Semang* also could be seen slightly in the Proto-Malay *Temuan*, Thai *Pattani* and two northern Malay sub-ethnic groups; *Melayu Kedah* and *Melayu Kelantan*. The Indian populations remained in the yellow cluster, although parts of their genome were also partitioned into the green component. At *K* = 4, all the Indian samples were assigned into a red colored cluster, which is exclusively separated from the yellow colored cluster of the non-African populations. It should be noted here that Indian proportions in the red colored fractions also appeared in three Peninsular Malaysia Malay sub-ethnic groups; *Melayu Kedah*, *Melayu Kelantan* and *Melayu Minang*, as well as in Thai *Pattani*. At *K* = 5, another cluster in pink color was apparent. This cluster mainly existed in the Proto-Malay *Temuan* and small proportions could be seen in both of the Chinese samples (*Jinuo* and *Wa*).

The structure of the Malays became apparent at *K* = 6, where a new cluster, denoted by the light blue color was confined mainly in the Peninsular Malaysia Malays, Thais and Indonesians samples. The light blue fractions are prominent in the Proto-Malay *Temuan* but rather slightly in the Chinese samples. Interestingly, the Indian components could be seen clearly in the samples of *Melayu Kedah*, *Melayu Kelantan*, *Melayu Minang*, and Thai *Pattani*. The yellow colored cluster mainly belonged to the Chinese samples although the proportions were also associated with the Malays, Proto-Malays, Thais and Indonesians indicating a common ancestor origin for these populations. The proportions of *Q* for each population in each of the six inferred clusters are shown in Table 2.

Higher K values revealed other clusters as shown in the *Q* plot at Figure 2. These clusters were generally confined to single populations; orange proportions in *Semang Jahai* for *K* = 7, while purple proportions mainly in Chinese *Jinuo* at *K* = 8. The splitting order of clusters varied greatly across different runs and different data sets. The newly derived clusters of *K* = 9 and *K* = 10 started to lose biological meanings as the real clusters and produced relatively lower *Q* proportions with unstable patterns in the graph of *Ln(Pr)*.

Nevertheless, the higher the number of *Ks*, the more it resembles or represent the modern genome of studied populations. As shown at *K* = 10 (Figure 2), the genetic structure in the genome of Malays appeared; 1) the *Melayu Bugis* were more delineate to Indonesian *Melayu* and *Toraja*, 2) the *Melayu Jawa* were similar to the Indonesian *Jawa*, with significant components of Chinese in their genomes, 3) the *Melayu Kelantan* and *Melayu Kedah* were more resemble to Thai *Pattani*, with significant admixture from Indian components.

### HS pattern in Malays SNPs genotype data

**Figure 3:**
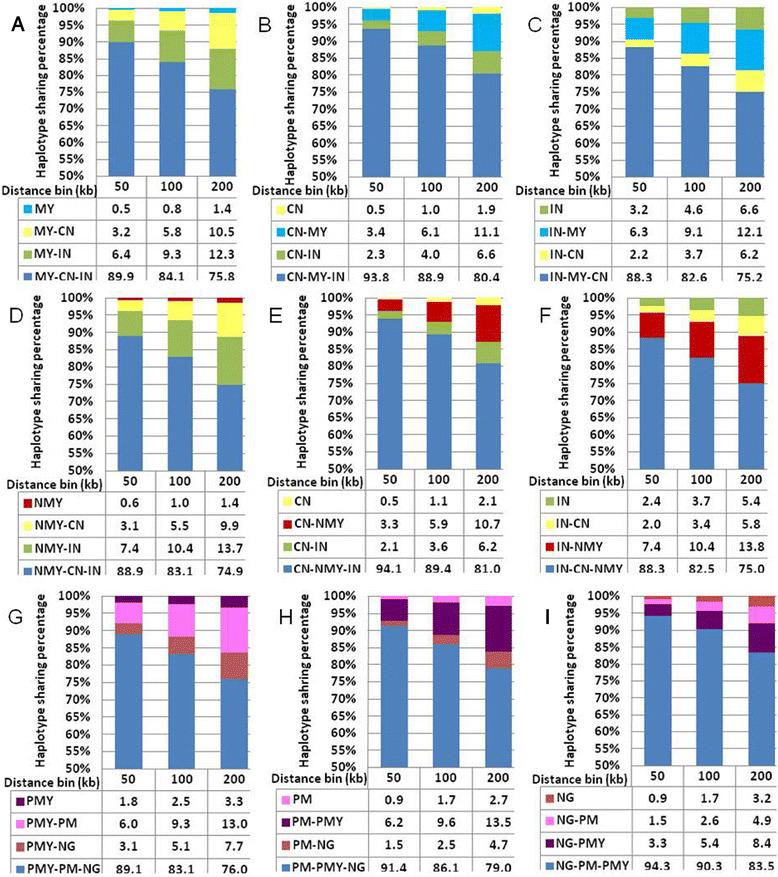
**Haplotype Sharing (HS) percentage of the Malays (MY), northern Malays (NMY), peninsula Malays (PMY), Chinese (CN), Indians (IN), Proto-Malays (PM) and** ***Semang*** **(NG).** Haplotypes in population A were identified by HSA as four classes: 1) private in population A; 2) shared with population B only; 3) shared with population C only; and 4) shared with all the three populations. **A)** MY; **B)** CN; **C)** IN; **D)** NMY; **E)** CN; **F)** IN; **G)** PMY; **H)** PM and **I)** NG. HS proportions were obtained by sampling 76 chromosomes 100 times without replacement and calculated without considering the frequencies of distinct haplotypes.

Partitions of the gene pool of Malays by HSAs were conducted in the framework of a three-population comparison as shown in Figure 3. The haplotypes of Malays (MY) were identified as four categories compared with Chinese (CN) and Indians (IN), that is, private in MY (MY), shared with CN only (MY-CN), shared with IN only (MY-IN), and shared with all three populations (MY–CN–IN). Generally, more than 98% of MY haplotypes could be found in either CN or IN with more contributions from IN populations. In the bins 50 kb to 200 kb, both MY (Figure 3A) and CN (Figure 3B) has less than 2% of private haplotypes, whereas IN have more than 3% of private haplotypes (Figure 3C). The same pattern also observed in population samples of northern Malays (NMY) (Figure 3D) that consist of *Melayu Kedah*, *Melayu Kelantan* and Thai *Pattani*. We further confirmed the HS pattern of peninsula Malays (PMY) data which comprised of all five Peninsular Malaysia Malay sub-ethnic groups including Thai *Pattani* compared with the *Orang Asli* Proto-Malays (PM) and *Semang* (NG). The PMY (Figure 3G) has slightly higher percentage of private haplotypes compared to both of the PM (Figure 3H) and NG (Figure 3I). All of these HS percentages were calculated without taking into account the frequencies of distinct haplotypes.

### STRUCTURE and HSAs phylogeny

**Figure 4:**
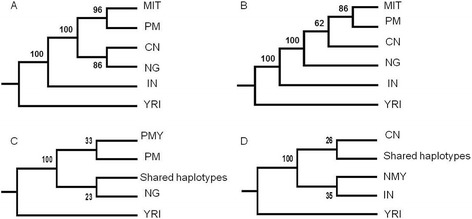
**Phylogenetic trees of STRUCTURE analyses and Haplotype Sharing Analyses (HSAs).** The phylogenetic trees re-constructed based on two types of genetic distance methods, which are **A)** Cavalli DC and **B)** Nei’s DA. The clade of MIT consists of Malays, Indonesians and Thais, PM is Proto-Malays, CN is Chinese, NG is *Semang*, IN is Indians and YRI is *Yoruba*. The phylogenetic trees based on haplotype sharing distance from 100 kb bins of HSAs were showed by; **C)** PMY is private haplotypes found only in Malays samples of peninsula; PM is private haplotypes found only in Proto-Malays samples; NG is private haplotypes found only in *Semang* samples; Shared haplotypes is found in all PMY, PM and NG samples. **D)** NMY is private haplotypes found only in northern Malay samples; CN is private haplotypes found only in Chinese samples; IN is private haplotypes found only in Indians samples; Shared haplotypes is found in all NMY, CN and IN samples; YRI is the African haplotypes that were used as outgroup.

The phylogenetic trees based on Cavalli DC and Nei’s DA genetic distances that were reconstructed using allele frequencies in each ancestral component inferred by Bayesian algorithm from the STRUCTURE analyses as well as the HSAs phylogeny are shown in Figure 4. The trees (Figure 4A and 4B) which reflected the identified ancestral clusters (*K* = 6) from STRUCTURE analyses consistently showed that the last split of branches was the clade of Malays, Indonesians and Thais (MIT), clustered together with PM. However, the trees revealed two slightly different topologies. The Cavalli DC showed simultaneously evolutionary divergence between the group NG and CN populations and the group of MIT and PM. Whilst, the topology of Nei’s DA support the subsequent divergence of evolutionary processes of the group of populations, which is more similar to the pattern of splitting orders in STRUCTURE analysis. In the phylogeny analysis of HSAs, Figure 4C showed that PM is a bigger haplotypes donor to PMY’s gene pool and much more related to PMY compared to NG. The HSAs phylogeny of the NMY (Figure 4D) has confirmed the closer genetic relationship with IN compared to CN, perhaps due to long-term admixture between both populations. These HSAs phylogeny patterns were concordance with the admixture analyses using the STRUCTURE program as described above.

## Discussion

Quantifying genetic distance is the main aspect in population genetic study, especially to characterize population structure and identify substructures (Lao et al. 2006; Rosenberg et al. 2002; Weir et al. 2005 and Tishkoff et al. 2009). Previous studies have also shown the importance of genetic distance assessments, such as in inference of migration patterns (Li et al. 2008; Ramachandran et al. 2005; Deshpande et al. 2009 and Laval et al. 2010) as well as in the need to adjust for population stratification in association studies Bryca et al. 2010; Patterson et al. 2010; Miclaus et al. 2009 and Li et al. 2010). This study was conducted to infer the population structure of Malays that may or may not have shared ancestry from other study populations. Therefore, the genetic distance based on Fst was chosen to measure the genetic variation distributions of studied populations. Fst is a powerful method to show population genetic structure by partitioning genetic variance within populations relative to between populations (Weir and Hill 2002; Weir and Cockerham 1984).

Many studies have shown that genetic distance of global populations correlates with geographic distance between populations, which refers to a situation called Isolation-by-Distance (IBD) (Li et al. 2008; Tishkoff et al. 2009; Ramachandran et al. 2005; Prugnolle et al. 2005 and Gonder et al. 2007). Nevertheless, based on the genetic distance analysis of this study, the IBD model can be applied to a particular group of populations, but not to the Malays group. The close genetic relationship between *Melayu Kedah* and *Melayu Kelantan* were mostly reflected to their geographic origin at the northern part of the peninsula, likewise Thai *Pattani* have smaller value of genetic distance to both of the northern Malays (Table 1). However, in the case of *Melayu Bugis* and *Melayu Jawa*, in which both population samples have been collected in the same state at the southern part of the peninsula, the result was in contrast with IBD. As shown in Table 1, the *Melayu Bugis* were genetically distant from other Malays but closely related to Indonesian *Toraja* from South Sulawesi, while the *Melayu Jawa* were significantly closer to Indonesian *Jawa* from Central Java. In this situation, the genetic distance corresponds largely to shared ancestry between populations, as been discussed in many previous studies (Hatin et al. 2011; Rosenberg et al. 2002; Jorde and Wooding 2004 and Consortium THP-AS 2009).

According to the history of Malay Peninsula and Indonesia, the migration of Indonesian people into mainland peninsula were very common whether due to trading activities or seeking asylum from civil war and colonial invasion (Taylor 2003; Sainuddin 2003 and Munoz 2006). For instance, the modern *Melayu Jawa* in peninsula are descendants of *Jawa* people that originated from Java Island. They migrated to the state of Johor and Selangor around the 15^th^ century to avoid conflicts due to civil war. The large scale migration of *Jawa* into peninsula was during British colonial era in between of 1880 to 1930 for seeking a better life from harsh invasion of the Dutch. The first influx of *Melayu Bugis* into Peninsular Malaysia from their origin in South Sulawesi was in the 17^th^ century. At that time, the Dutch were expanding their trade and political control on Sulawesi. Arising conflicts between the colonial and colonized people were inevitable and most of the *Bugis* people migrated to Johor state at the southern part of the peninsula. Later on, they also settled in Selangor state for over a few centuries. Since then, the *Melayu Bugis* and Indonesian *Toraja* which originated from the same geographic origin were geographically separated for hundreds of years.

It is also known from the history and anthropological evidence, the *Melayu Minang* were originally *Minang kabau* people from West Sumatra. They migrated to Malay Peninsula in the 14^th^ century, long before the arrival of *Bugis* and *Jawa* people. They started the colonization of Negeri Sembilan, a state in the middle of the western part of Peninsular Malaysia and at present day are known as *Melayu Minang*. However, in relation to the other Malays, the *Melayu Minang* were genetically closer to both of *Melayu Kedah* and *Melayu Kelantan*, than to *Melayu Jawa* (Table 1). Here, the genetic distance between those Malay sub-ethnic groups may have been affected by the presence of genetic admixture in their genetic data from the same mixing populations. In quantifying genetic distance among populations that experienced admixture from the same mixing populations, interpreting the relationships can be tricky as the admixture reduces the average of genetic distance between them (Handley et al. 2007; Halder et al. 2008; Auton et al. 2009 and Moorjani et al. 2011).

It is interesting to see the close genetic relatedness among Malays (except for *Melayu Bugis*) with the Proto-Malay *Temuan*, Indonesian *Jawa* and Chinese *Wa* from Yunnan, China. This may imply a common origin for all of those populations regardless of their historical migration patterns as been reported (Bellwood 1997 and Omar 2004). But it is more intriguing to see how genetically close the *Melayu Kedah* and *Melayu Kelantan* are to the Indian *Telugu* and *Marathi* compared to any other populations in this study. This result has conformed to the historical contact between those populations with the Indians (Arasaratnam 1970).

Apart from being close to Proto-Malay *Temuan*, the *Melayu Kedah* and *Melayu Kelantan* also showed relatedness to the earliest aboriginal people of peninsula in view of having the smallest value of genetic distance to both of the *Semang* sub-groups, *Jahai* and *Kensiu* compared with any other Malays. The Indonesian *Jawa* and Chinese *Wa* also shared the same lower value to both of the *Semang*, compared to other group of studied populations. This may imply two probable situations that could explained the genetic relatedness of those populations. Firstly, a common origin for all of those populations with subsequent evolutionary divergence due to pre-historical migration and become closer with subsequent recent migration as been implied by the HUGO-PASNP Consortium (Consortium THP-AS 2009). Secondly, they might have diverse origin from simultaneously pre-historical evolutionary divergence (Cavalli-Sforza et al. 1994) but admixed due to recent migration patterns as been explained by (Andaya 2001). To be certain about which are the most favorable demographic histories among them, further analysis was conducted in model-based approach using ancestral components that will be discussed subsequently.

Meanwhile, the MDS has been widely used for the analysis of proximity data among objects to reveal the hidden structure underlying the data (Steyvers 2002; Borg and Groenen 2005) especially in DNA microarray data (Tzeng et al. 2008). In this study, the genetic structure of Malays showed by Fst was successfully recapitulated in two and three dimensions (2D and 3D) models as shown in Figure 2. The Malay populations are shown explicitly as three sub-clusters on both of the 2D and 3D platforms, signifying an existence of substructure within the Malays. This could be achieved by finding the disposition of studied populations that are compatible with the given genetic distances among them on a map. The Euclidean distance is used to represent the transformed data in such a way that the MDS clustering matches the original data as much as possible even in a smaller number of dimensions (Borg and Groenen 2005). However, the distance-based method could not provide the ancestral membership coefficients of the admixture among the populations. Hence, we implemented further analysis to determine the genetic admixture and ancestry of the Malays.

Previous studies have shown the robustness of the STRUCTURE software in inferring the population structure and ancestry in variety of population data (Xu et al. 2010; Consortium THP-AS 2009; Bamshad et al. 2003; Rosenberg et al. 2005 and Witherspoon et al. 2007). In this study, the assignment of individuals into inferred clusters was in accordance to historical and demographical background of studied populations. At *K* = 2, all individuals of the 17 pre-defined populations was predominantly separated into African and non-African ancestries, indicating that the modern human dispersal originated from Africa (Cavalli-Sforza et al. 1994; Bowcock et al. 1994). The emergence of cluster that is specific to *Orang Asli Seman*g of Malay Peninsula as early as at *K* = 3, supports the arrival of the *Semang* into SEA region via the first wave from Africa as postulated by many researchers (Allen 1879; Carey 1976; Fix 1995; Consortium THP-AS 2009; Kashyap et al. 2003 and Hill et al. 2006). The fact that their cluster occurred before the Indian cluster at *K* = 4, may be caused by the great effect of genetic drifts in their genetic data due to population bottleneck event and later exhibit the founder effects. As they are extremely geographically isolated and conserved from the outside world, they have preserved their ancestral allele state since the divergence. This is unlike the Indians, who have widespread admixtures with Europeans (Brahmachari et al. 2008). Histories of language shifting are also common in some of the Indian populations, as shown by the Indian *Marathi* in this analysis.

It is also noted that the ancestral component of *Semang* could be seen in Indians, Proto-Malays, Thai *Pattani* and both *Melayu Kedah* and *Melayu Kelantan*. However, based on the *Q* proportions of *K = 6* as the ancestral cluster, the component was not significantly high with merely 0.05 in *Melayu Kelantan* and Proto-Malays, whilst much lower in the other Malays. Interestingly, the admixture coefficients of the Indian ancestral component exist in both of the *Melayu Kedah* and *Melayu Kelantan*, as well as in *Melayu Minang* and Thai *Pattani*. The highest coefficients were in *Melayu Kedah* with 0.21, followed by *Melayu Kelantan* 0.16, Thai *Pattani* 0.14 and *Melayu Minang* 0.12.

In population HSAs, the Indians have higher percentage of population private haplotypes than the Chinese and the Malays. This pattern is more compatible with the scenario of population admixture in Indians. However, high level of haplotype diversity is not just expected in an admixture population with divergent ancestries, but also in an ancient population as it has long time to accumulate much more private haplotypes. The HS pattern of Indians with the Chinese and Malays was relatively lower than the HS of those two groups with each other. The reason that could cause to these HS pattern perhaps due to the main separation between Indians and Asian populations dates to about 60,000 years ago (Cavalli-Sforza and Feldman 2003) while the populations in Southeast Asia and East Asia (China) have very close connections in the more recent past, either due to Neolithic expansions from China into mainland Southeast Asia and Island Southeast Asia or somewhat earlier migrations in the late Pleistocene or Early Holocene due to climate change and sea-level changes (Ricaut et al. 2006; Bellwood and Dizon 2008 and Hung 2008).

The STRUCTURE results exhibited very close estimates with the HSAs results, suggesting major contribution of Indian haplotypes in the northern Malays. The centralization of the ancient Indianized kingdoms had occurred in mainland Southeast Asia such as Thailand, Cambodia, and Myanmar for centuries in the early millennium (Tarling 1999; Stark 2006). Although Hinduism also existed in some of the Indonesian islands (eg. Sumatra and Java Island), it was more predominant among the populations in mainland region and the northern part of Malay Peninsula (Shuhaimi 1984; Allen 1997 and Syukri 2002). The existence of Indian ancestral component within these northern Malay populations is relevant to their early historical contacts with the Indians. The long-term historical contacts between Malays and Indians, may explain the higher admixture coefficients in both *Melayu Kedah* and *Melayu Kelantan*.

Furthermore, the existence of the Indians haplotypes in the gene pool of the northern Malays may signify that they are the oldest Malay populations in Malay Peninsula as the Indians had been conspicuous in the region very much earlier, since the proto-historic times. The ancient Hindu Malay kingdoms which arose approximately in 100 before common era (BCE) to 7^th^ century CE such as *Chi Tu, Langkasuka* and *Kadaram* have controlled much of the northern Malay Peninsula (Arasaratnam 1970). The Indian influxes continued to expand during the subsequent empires of *Srivijaya* and *Majapahit* (Paul 1961). These early Malay states were heavily influenced by concepts of religion, government and arts that were brought by the Indians. The proto-historic of Malay Peninsula ended in the beginning of 15^th^ century CE with the emergence of Malacca Sultanate. Malacca that encompassed most of modern day Peninsular Malaysia, Singapore and a great portion of eastern Sumatra thrived into the most important entrepôts in Southeast Asia and a hub of Islamic studies, spreading Islam to Malay Archipelago in 16^th^ century CE. Still, traces of the Indian influence can be found in Malay culture until today (Arasaratnam 1970; Shuhaimi 1984 and Syukri 2002).

Possible admixture between Malays and Indians could also have occurred during the British colonial period from the 19th to the middle of the 20th century. However, the Indians are not a large component of the Kedah and Kelantan population either during or after the British colonial era as most of them reside in the western and north-western regions of Peninsular Malaysia which are the location of the big cities and large urban areas in the country. In the Kelantan state which is the origin of the *Melayu Kelantan*, the total population is about 1.67 million and the percentage of the Indian community is only 0.2% of the population. In the Kedah state which is the origin of the *Melayu Kedah*, the total population is about 2.04 million and the percentage of the Indian community is also slightly higher than in Kelantan with 6.6% of the population (Hunley et al. 2009). Moreover, the sampling procedure stringently followed the inclusion and exclusion criteria that emphasized the three generations without any different ethnic admixture rule for an individual to be considered as a valid subject for this study. Hence, we believed that the admixture in both *Melayu Kedah* and *Melayu Kelantan* with Indians was ancient and has occurred during the early existence of the Malays.

The ancestral component of Proto-Malay *Temuan* appeared at *K* = 5, while the ancestral component of Malays emerged at *K* = 6 and both of the components also existed in the Chinese individuals, especially in Chinese *Wa* at respective clusters. In those inferred clusters, the ancestral component of Chinese (yellow component) was predominant in all the Malays, Thais, and Indonesians as well as in Proto-Malays itself. This is in accordance to the historical and anthropological evidences of the migration pattern of Proto-Malays from Yunnan, southern mainland China (Bellwood 1997; Carey 1976). The yellow component also might be related with a large Neolithic input from China into mainland and island Southeast Asia due to the expansion of agriculture and animal domestication (Bellwood and Oxenham 2008; Bellwood 2011). Although the Austronesian dispersal did not originated from the early farming dispersal, but it was a peripheral result of the demographic impetus and technological advancement by the developments of food production in mainland East Asia (Bellwood 2011). Furthermore, the peopling of pacific region started from earlier migration of modern human expansion from Africa throughout much of Southeast Asia during a period of relatively stable climate and sea-level from 45,000 year before present (YBP) to 40,000 YBP. The extreme climate and rapidly changes of sea-level during the Last Glacial Maximum lead to decrease the expansion of human populations from 33,000 YBP – 16,000 YBP (Bird et al. 2004; Forster 2004). Later, the post-glacial expansion in coastal settlement arose concurrently with the development of coastal ecosystems and environments due to the slow rise of the sea-level. However, the sea-level fluctuations inhibited the coastal settlement and the drop in sea level in the mid-Holocene may have caused widespread human expansion throughout Oceania (Bird et al. 2004; Pope and Terrell 2008). The yellow component suggests that despite having great admixture or genetic differences due to genetic drift, all of these populations have a common ancestor, which is referred to as Southern Mongoloid group of races.

In relation to the modern Malays, it is known that Malays have been previously referred to as admixed Deutero-Malays, which are the descendants of the Proto-Malays who had admixture with other populations, such as Arab, Sumatran and Siamese (Sainuddin 2003). Other sources have postulated that the Deutero-Malays originally migrated through the southern part of China, and reached the Malay Peninsula about 1500 to 2000 years ago, after the arrival of the Proto-Malays (Fix 1995). According to Kasimin (Kasimin 1991), compared with the arrival of Indians and Chinese to Malay Peninsula, the Deutero-Malays were the earliest to be settled. Then, the vast and subsequent influxes of other populations to peninsula, mainly due to trading activities had integrated the Deutero-Malays into admixtures. These Deutero-Malays are known as the present day Malays. Given the vague historical facts on the origin of Malays with a fine line to differentiate between Proto-Malays and Deutero-Malays, it is still evident that these populations have profoundly close genetic relationship and shared a common ancestor with the Chinese.

The ancestral clusters or the most biologically sensible number of clusters which captured the structure of the given genotyped data was identified as six clusters (Table 2). In this ancestral clusters (K = 6), any pre-defined populations which shared similar distinctive *Q* values were merged into the inferred cluster. These clusters associate mainly to self-identified geographic origin or geographic proximity, linguistics family and their ancestry. The first cluster is referred as the cluster of Austronesian speakers and includes all the Malays, Thais and Indonesians. The second cluster was exclusive to Proto-Malays ancestry, which is *Temuan* in this study. The fact that these Proto-Malays are different from the other Austronesian speakers although belonging to the same linguistic family, perhaps due to evolution forces such as genetic drift or selection pressures that have reshuffled their genetic components. Yet, both these clusters still have conserved great proportions of Chinese ancestral component, testifying their Southern Mongoloid morphological features.

The third cluster belongs to the Austro-Asiatic speakers, which are the *Semang Jahai* and *Kensiu*. The fourth cluster associates with Chinese ancestry and belongs to the Chinese *Jinuo* and *Wa* from Yunnan, China. However, these Chinese populations speak different languages; *Wa* speak Austro-Asiatic whilst *Jinuo* speak Sino-Tibetan language. These two populations are the indigenous populations in Yunnan and as shown by the HUGO-PASNP Consortium (Consortium THP-AS 2009), both populations were clustered among indigenous Thais populations who also speak Austro-Asiatic language. This indicates a history of language shifting in the Chinese *Jinuo* (Dutton and Tryon 1994). Although sharing the same linguistic family, a great difference between Chinese *Wa* and the *Semang* group in Malay Peninsula is likely due to their historical divergence with wide range of demographic histories (Consortium THP-AS 2009). The fifth cluster belongs to Indian ancestry, consist of *Marathi* and *Telugu*. Again, despite having different linguistic family, both the Indians were clustered in the same ancestral cluster. Lastly,the sixth cluster consisted of only *Yoruba* with African ancestry who speak Niger-Congo language.

The population genetic structure of Malays and other studied populations became more apparent at higher number of clusters, but it also might not representative of anything and just residuals of the methodology. Thus, the interpretation must be made carefully as well as must be supported by other evidence, either historical or anthropological. In this analysis, it is clear that whole-genome data of *Melayu Bugis* were more delineated to Indonesian *Melayu* and *Toraja* from Indonesia with very minimal admixture. The *Melayu Jawa* were similar to the Indonesian *Jawa*, with significant components of Chinese in their genomes. The *Melayu Kelantan* and *Melayu Kedah* resemble more to Thai *Pattani*, with significant admixture from Indian components. Although the *Melayu Minang* also shared the Indian ancestral component, they were slightly different at this higher level of clusters as they exhibit almost none of the Chinese ancestral component. This may be due to different demographic histories compared to other Malays as has been reflected by their unique maternally cultural and traditional rule, called “*Adat Pepatih*” (Reid 2001; Ricklefs 2001). This unique sociological cultural may have had greater effect of pressure selections in the *Melayu Minang* population. The result of admixture analysis was concordance with the previous study of human leukocyte antigen (HLA) polymorphism and population structure analysis of Malays (Hatin et al. 2011; Edinur et al. 2009).

The resulting *Q* plot from STRUCTURE and HSAs partition plots did not revealed the relationship among the components in term of evolutionary history. Thus, phylogenetic trees were reconstructed to further refine the analysis. The topology of tree produced by Cavalli DC contradicted most known patterns of historical migrations. Based on historical facts, it is unlikely that Malays and Proto-Malays have a simultaneously historical divergence with the *Semang* group. Many evidences have shown that the original inhabitants of the Malay Peninsula are the *Semang*. For instance, the oldest Paleolithic human skeleton estimated about 11,000 years old, was reported to have genetic similarities with the *Semang* (Majid 2005). The other topology, which was produced by Nei’s DA is more favored to reflect the evolution histories among the study populations. Previous genetic studies have postulated the northwards migration of SEA people to central and eastern Asia (Consortium THP-AS 2009; Su et al. 1999) before gradually migrated back southwards (Rahman et al. 1998; Andaya 2001; Omar 2004; Bellwood 1997 and Fix 1995). The phylogeny analyses of HSAs indicated greater similarity where the Malays are more related to Proto-Malays than to *Semang*. Although the ancestry line of Malays were traced back to the Proto-Malays and the Chinese, the Indians have contributed more haplotypes to the northern Malays that may resulted to the *Melayu Kedah* and *Melayu Kelantan* to be genetically different from the other Malays.

## Conclusions

The genetic clustering by model-based approach has successfully showed the admixture and ancestral coefficients within the studied populations that is in line to the historical backgrounds, which cannot be achieved by the distance-based method. The need to characterize the genetic make-up of this admixture proportions, especially in genetic-based medical studies is very important as it clearly affect the gene pool of a particular population. Thus, this study suggests that a larger scale research of targeted admixed populations on other ethnic groups in Malaysia should be conducted in the near future.

## Methods

### Population samples and genotype data

All genotype data were generated from DNA samples that were collected with informed and written consent and approved by local ethics committees in Malaysia (Research and Ethics (Human) Committee, School of Medical Sciences, Universiti Sains Malaysia (USM) and Medical Ethics Committee, Pusat Perubatan Universiti Malaya (PPUM)), China (Ethical Committee, Chinese National Human Genome Centre (CNHGC) at Shanghai, PR China), Indonesia (Research Ethics Commission, Eijkman Institute for Molecular Biology, Indonesia), India (Human Ethics Committee, Institute of Genomics and Integrative Biology (IGIB)) and Thailand (Ethics Committee Faculty of Medicine, Prince of Songhkla University, Thailand.

**Figure 5:**
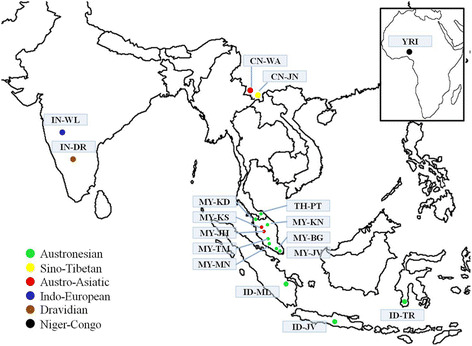
**Map of the Asian continent depicting geographic locations of the sampled populations in six countries.** The small box on the upper right corner of the figure shows African continent. The colors denoted the linguistic family of the populations.

In this study, samples were carefully selected by the inclusion and exclusion criteria that emphasized the three generations without any different ethnic admixture. All datasets used in the study were derived from 17 populations representing six linguistic families that consisted of 472 unrelated individuals from five Malay sub-ethnic groups (*Melayu Bugis*, *Melayu Jawa*, *Melayu Minang*, *Melayu Kedah*, and *Melayu Kelantan*); three *Orang Asli* sub-groups (*Jahai*, *Kensui* and *Temuan*); one Thai population (*Pattani*), three Indonesian populations (*Melayu*, *Jawa* and *Toraja*); two from Yunnan, China (*Jinuo* and *Wa*); two from India that were assigned based on their language (*Telugu* and *Marathi*); and one from Nigeria, Africa (*Yoruba*). The map of the Asian continent depicting geographic locations of the sampling populations is shown in Figure 5. All samples were assigned anonymously and code identified at analysis and data point as shown in Table 2.

The Affymetrix GeneChip Mapping Xba 50 K Arrays were used to genotype single nucleotide polymorphisms (SNPs) on a genome-wide scale for 109 unrelated individuals of the five Peninsular Malaysia Malay sub-ethnic groups and one Thai *Pattani*. Meanwhile, the additional genotype data of 363 unrelated individuals from other 11 populations were obtained from the database of the Pan Asian SNP Initiative (PASNPI) Consortium, except for *Yoruba* that were obtained from the International HapMap Consortium.

A total of 58,960 SNPs that have been genotyped for all the sampled individuals were screened under the strict criteria of data quality control. Samples with a call rate below than 90% were excluded from further analysis. A total of 4,166 SNPs (7%) were filtered out (Unmapped to Affymetrix annotation file, chromosome X SNPs and intersection SNPs with downloaded Pan-Asian SNP genotypes), leaving a total of 54,794 autosomal SNPs as the final genotype data for each individual to be used in further analyses.

### Genetic differentiation between populations

The genetic divergence between studied populations were determined by Fixation Index Statistic (Fst) as described by Weir and Hill (Weir and Hill 2002). Package for Elementary Analysis of SNP data v1.0 (PEAS) (Xu et al. 2010) was used to calculate allele frequency and genetic distance between populations. The genetic structures of population were assessed by a multivariate statistical technique such as MDS analysis using SPSS 18. The input data was the genetic distance matrix of pair-wise Fst. In this analysis, the convergence of the S-Stress value was set to 0.001 and the iterations were set to a maximum of 30. The number of dimensions employed was two dimensions and then increased to three dimensions.

### Admixture analyses by STRUCTURE

We used STRUCTURE v2.3.3 (Pritchard et al. 2000), a model-based clustering software which implements the Markov Chain Monte Carlo (MCMC) algorithm within a Bayesian framework to estimate the genetic structure and distribution of ancestral component for each individual of studied populations. The admixture model and correlated allele frequencies between populations in the parameter setting of STRUCTURE analysis are powerful to detect subtle population genetic structures, such as in highly admixed populations (Falush et al. 2003). It assigns individuals into pre-specified clusters (*K*) with estimated membership coefficient (*Q)* for each cluster which were fitted with posterior probabilities of *Pr (X|K)* solely based on the given genotyped data *(X)* without incorporating any other population information (Pritchard et al. 2000; Falush et al. 2003). The value of *K* that maximized the value of *Pr (X|K)* which showed by the graph of *Ln(Pr)* over the run of analysis is the most probable number of ancestral clusters (Pritchard et al. 2003).

We created sub-datasets from the full SNPs dataset using between marker distance (BMD) as the threshold value in the re-sampling procedures and a total of five sampling datasets (S1-S5) have been produced. The average of the BMD is 550 kb and each dataset contain approximately 3700 number of SNPs that were evenly distributed across 22 autosomal chromosomes. The sub-datasets were created because of STRUCTURE’s limitation which does not deal with strong background linkage disequilibrium in the data (Falush et al. 2003). The used of sub-datasets also worth to cut the analysis time due to the computational intensity of the STRUCTURE analysis that is time consuming (Falush et al. 2003).

We ran a series of analysis in STRUCTURE from *K* = 2 to *K* = 10 and number of iterations were set to 10 times for each *K*s and each datasets in order to verify the consistency of the results. Hence, we have submitted a total of 450 running analyses (5 sub-datasets × 9 inferred cluster x 10 iterations = 450) with 30,000 burn-in length and 20,000 MCMC iterations in each analysis to STRUCTURE software. The distribution of the alpha parameter showed a relatively constant distribution indicating convergence after 20,000 iterations. The estimated *Q* matrices from the STRUCTURE outputs were carefully observed and compared using the symmetric similarity coefficients (SSC) and there were no big differences in the estimation of *Q*s for all runs. The SSC was computed via permutation analyses of *Q* matrices for any number of clusters from multiple runs or multiple datasets generated by STRUCTURE software. The analyses were implemented by Cluster Matching and Permutation Program (CLUMPP) (Jakobsson and Rosenberg 2007). A program called *distruct* (Rosenberg 2004) was used to provide much finer control of the graphic plot of *Q*. It displays each individual as a line segment that partitioned into *K* colored components, which represent the individual’s *Q* in the *K* clusters.

### Haplotype-sharing analyses (HSAs)

The fast PHASE v1.2 (Scheet and Stephens 2006) was used to estimate haplotypes for each individual from 54,794 SNPs data. The number of random starts of the EM algorithm (−T) was set to 20, and the number of iterations of EM algorithm (−C) was set to 50. The software provides an estimation of the true underlying patterns of haplotype structure and to enhance the accuracy of the analysis, population labels were applied during the model fitting procedure (Scheet and Stephens 2006). The percentages of haplotype sharing (HS) among populations were determined based on (Xu et al. 2009) by HaploSharing program of the PEAS v1.0. The analysis binned the inferred haplotypes within particular size of windows and let a window slide by half of the other window size each time, considering the substantial variation of recombination across human genome (The International HapMap Consortium 2007; Li et al. 2008).

In this study, we adopted three sizes of sliding window (50 kb, 100 kb and 200 kb) to estimate the HS in three-population framework. According to (Xu et al. 2009), if there were three populations, A, B, and C, the haplotypes of one population can be identified as four categories when compared with those of the other two populations, regardless of the haplotype frequency. For instance, the haplotypes of population A are classified into four haplotype categories: 1) haplotypes are private in population A (denoted by H_AP_), 2) haplotypes are common in populations A and B but not in population C (H_AB_), 3) haplotypes are in common in populations A and C but not in population B (H_AC_), and 4) haplotypes are common in all populations (H_ABC_). The haplotypes for populations B and C can be similarly defined.

In the HSAs of this study, the particular populations were merged into a group pursuant to the result of STRUCTURE analysis. For the first HSA, all Peninsular Malaysia Malays, Thai *Pattani* and Indonesians were merged into a group named Malays (MY), the Chinese (CN) group consisted of *Jinuo* and *Wa*, while the group of Indians (IN) comprised of *Marathi* and *Telugu*. This analysis was done to indicate the proportion of HS among the three groups and to identify which group was a bigger genetic contributor to Malays group. To represent the divergence pattern of the northern Peninsular Malaysia Malays, we combined the *Melayu Kedah*, *Melayu Kelantan* and Thai *Pattani* into a group named northern Malays (NMY) and implemented the second HSA with the Chinese and Indians groups. The third HSA was done to examine the relationship of the peninsula Malays (PMY) group consisted of Peninsular Malaysia Malays and Thai *Pattani* with the indigenous populations, which are the *Orang Asli Semang* (*Jahai* and *Kensiu*) grouped as Negrito (NG) and the Proto-Malays *Temuan* labeled as Proto-Malays (PM).

However, the varying sample size among populations could affect the HS results. Taking this point into consideration, we performed a procedure called Non-Replace Sampling (NRS) in the HaploSharing program. The number of haplotypes in each genomic window on 76 chromosomes (38 individuals is the minimal sample size in this study) were counted for each population. The bootstrap replicate for the sampling procedure was 100 times and the results were averaged for each window. Any haplotype that observed less than twice in this analysis was excluded.

### Phylogeny analyses

The phylogenetic trees of HSAs were based on four-population framework of HS estimation as African *Yoruba* (YRI) samples were added to serve as an outgroup for the rooted trees. The haplotype sharing distances among the group of populations from 100 kb bins were calculated based on Fst (Weir and Hill 2002). The distance based population trees were reconstructed using the Neighbor Joining algorithm (Saitou and Nei 1987) by Molecular Evolutionary Genetics Analysis 4 (MEGA) (Tamura et al. 2007) and two programs from Phylogenetic Inference Package 3.67 (Phylip) (Felsenstein 2007) which are Neighbor and Consense. The phylogenetic trees of STRUCTURE analyses based on Bayesian algorithm were reconstructed using the estimated allele frequencies of the inferred clusters. The PEAS program called *ClusterDis* was used to calculate two types of genetic distances among the inferred clusters, named Cavalli-Sforza Chord Distance (Cavalli DC) (Cavalli-Sforza and Edwards 1967) and the Nei’s Matrix Distance (Nei’s DA) (Nei et al. 1983). Bootstrapping test was performed for 1000 times and the phylogenetic trees were rooted using YRI, assuming that the exit point of human diaspora in Africa was correct.

## Abbreviations

MDS: Multi-dimensional scale
PASNPI: Pan Asian SNP Initiative
MIT: Malays, Indonesians and Thais
IBD: Isolation-by-distance
BCE: Before common Era
YBP: Year before present
HLA: Human Leukocyte Antigen
USM: Universiti Sains Malaysia
PPUM: Pusat Perubatan Universiti Malaya
CNHGC: Chinese National Human Genome Centre
SNPs: Single nucleotide polymorphisms
CLUMPP: Cluster matching and permutation program
SSC: Symmetric similarity coefficients
BMD: Between marker distance
MCMC: Markov Chain Monte Carlo
UKM: Universiti Kebangsaan Malaysia
UMBI: Medical Molecular Biology Institute
2D: Two dimensions
3D: Three dimensions
HS: Haplotype sharing
MY: Malays
NMY: Northern Malays
PMY: Peninsula Malays
CN: Chinese
IN: Indians
PM: Proto-Malays
YRI: African yoruba
NG: Semang
HSAs: Haplotype sharing analyses
NRS: Non-Replace Sampling
NG: Negrito
